# IRON DEFICIENCY ANEMIA AND ITS RELATIONSHIP WITH SOCIOECONOMIC VULNERABILITY

**DOI:** 10.1590/1984-0462/2020/38/2019031

**Published:** 2020-06-05

**Authors:** Élida Mara Braga Rocha, Amanda Forster Lopes, Silvia Maira Pereira, Claudio Leone, Luiz Carlos de Abreu, Patricia Dore Vieira, Sophia Cornbluth Szarfarc

**Affiliations:** a Faculdade de Juazeiro do Norte, Juazeiro do Norte, CE, Brazil.; b Institute of Health and Biotechnology, Universidade Federal do Amazonas, Coari, AM, Brazil.; c Department of Nursing and Nutrition, Universidade de Taubaté, Taubaté, SP, Brazil.; d School of Public Health, Universidade de São Paulo, São Paulo, SP, Brazil.; e Universidade Federal do Espírito Santo, Vitória, ES, Brazil.; f School of Medicine, Estácio Juazeiro Norte, Juazeiro Norte, CE, Brazil.

**Keywords:** Anemia, iron-deficiency, Child, preschool, Health status disparities, Health vulnerability, Child, Anemia ferropriva, Pré-escolares, Desigualdades em saúde, Vulnerabilidade em saúde, Criança

## Abstract

**Objective:**

To analyze the association of anemia with the socioeconomic vulnerability of preschoolers in public day care centers, in the city of Taubaté, SP, Brazil.

**Methods:**

This is a cross-sectional study with a probabilistic sample that analyzed 363 children assisted in public day care centres in low-income and high-income areas of Taubaté, SP, Brazil. The hemoglobin concentration (Hb), dependent variable, was obtained by digital puncture, considering anemic children with Hb concentration <11.0 g/dL. The independent variables such as socioeconomic and demographic conditions were collected by a semi-structured questionnaire.

**Results:**

The prevalence of iron deficiency anemia was 19.3% among preschoolers. Children from day care centers with high socioeconomic vulnerability had lower Hb concentration than those from a non-vulnerable area (p<0.05).

**Conclusions:**

The lower concentration of hemoglobin in preschoolers is associated with the location of day care centers in an area of socioeconomic vulnerability. Children attending these day care centers face adverse family conditions such as low income, working mothers, and mothers with low schooling, though they receive social benefits and monitoring by public health services.

## RESUMO

**Objetivo:**

Analisar a associação da anemia com a vulnerabilidade socioeconômica de pré-escolares de creches públicas da cidade de Taubaté, SP, Brasil.

**Métodos:**

Trata-se de um estudo transversal com amostra probabilística que analisou 363 crianças assistidas em creches públicas de regiões de baixa e alta renda de Taubaté. A concentração de hemoglobina (Hb), variável dependente, foi obtida por meio de punção digital, considerando anêmicas as crianças com concentração de Hb<11,0 g/dL. As variáveis independentes como condições socioeconômicas e demográficas foram coletadas por questionário semiestruturado.

**Resultados:**

A prevalência de anemia por deficiência de ferro foi de 19,3% entre os pré-escolares. As crianças das creches de elevada vulnerabilidade socioeconômica apresentaram concentração de Hb estatisticamente menor do que aquelas de região não vulnerável (p<0,05).

**Conclusões:**

A menor concentração de Hb em pré-escolares está associada à localização das creches em região de vulnerabilidade socioeconômica, uma vez que as crianças que frequentam tais creches enfrentam condições adversas familiares, como baixa renda, mães que trabalham e com baixa escolaridade, embora recebam benefício social e acompanhamento pelos serviços públicos de saúde.

## INTRODUCTION

Iron deficiency anemia in children under five years of age stands out among specific nutritional deficiencies, with a prevalence of 42.6%, affecting 273.2 million children worldwide. Its deleterious consequences, which are often irreversible in adulthood, include a population that is physically disabled and not prepared for the labor market, often being socially excluded.^1^ ^,^ ^2^ ^,^ ^3^

It is noteworthy that Brazilian preschoolers and women of reproductive age, the populations that are most sensitive to anemia, continue to show high prevalence of this nutritional deficiency despite interventions for flour fortification and iron supplementation, which were implemented over a decade ago.^4^ ^,^ ^5^ This statement is confirmed by the high prevalence of anemia described in the National Demography and Health Survey in 2006, which identified anemia in 20.9% of Brazilian children and 29.4% of Brazilian women, representing a moderate risk situation in public health.^6^

In this sense, children, being dependent on the environment and susceptible to psychological, socioeconomic and cultural changes, are part of the most significantly relevant group, since their resilience to different vulnerability scenarios is investigated to understand various public health problems. In this way, the situations of social inequity and inequality, expressed through potential for illness, or non-illness,^7^ overcome the probabilistic character of risk by pointing out a set of aspects that go beyond the individual, covering collective aspects of biological, psychological, cultural, economic and political order, which lead to susceptibility to diseases or injuries.^8^

The dietary deficiency in sources of iron is the major determinant of anemia, but iron deficiency has its origin in a broad context strongly linked to socioeconomic and cultural factors. Developing countries are marked by adverse social conditions and inefficient public policies that cause health inequities and, therefore, translate into different risks for the disease, causing unnecessary suffering to the population.^9^

Thus, the recognition of health needs of individuals and families from different social groups is of great importance in identifying the profiles of social reproduction and health-disease, allowing the control and monitoring of public health,^10^ especially when it involves nutritional problems among children, an issue that high, middle and low income countries alike are facing. Therefore, this article aimed to analyze the association of anemia with the socioeconomic vulnerability of preschoolers in public day care centers in a city in the Paraíba Valley, São Paulo, Brazil.

## METHOD

Cross-sectional study with preschoolers aged 24 to 48 months who attended municipal day care centers in Taubaté, São Paulo, in 2014. Taubaté is located in the metropolitan region of the Paraíba Valley and on the state’s North Coast, 123 km from the city of Sao Paulo. It has a high human development index (HDI), being among the 40 best municipal HDIs in the country. This is justified by the gross domestic product (GDP) per capita, which in 2011 was one of the highest among those described for the São Paulo municipalities. This is in addition to the good basic sanitation indicators, the high percentage of public educational coverage and the various Family Health Strategy (FHS) teams, as well as the traditional basic health units with high population coverage distributed throughout the municipality.

Based on the Occupation, Income and Education Survey, the city of Taubaté was divided into five regions^11^ and subsequently grouped into two socioeconomically distinct groups: vulnerable region, with an average family income of 1.35 minimum wages (class E); and non-vulnerable region, with average family income between 2.8 and 11.3 minimum wages (classes A, B, C and D). The sample size was calculated based on the assumption that the difference in hemoglobin concentration (Hb) between children in the vulnerable and non-vulnerable regions was equivalent to 1/3 standard deviation from the average Hb of the healthy population. Adopting α=5% and β=20%, at least 145 children would be needed for each region. Considering the 20% increase for eventual losses, the final sample was estimated at 350 children. The sampling was probabilistic, with successive random draws of day care centers in the two regions until the total sample was completed.

Regardless of the region where the day care centers are located, there are two types of pre-school care regime in the municipality, part-time and full-time. Students enrolled in the part-time program receive two meals a day at the day care center: preschoolers in the morning period were offered breakfast and lunch; and those in the afternoon period were offered an afternoon snack and dinner. For children who stayed full-time, four complete meals were offered: breakfast, lunch, afternoon snack and dinner. It is worth noting that the menu was planned in the same way for all public day care centers in the municipality, in accordance with the rules of the Department of Education of the City of Taubaté, being carried out daily in a proper environment inside the day care center facilities.

The diagnosis of anemia was made by measuring the Hb concentration. A capillary blood sample was obtained by puncture with a disposable lancet, puncturing it in the fingertip pulp of the middle or ring finger of the child’s left hand. For the measurement of Hb, the second drop of blood was collected by means of a fixed-volume micropipette, totalling 0.1 ml of blood, which was then added to 1.5 ml of Drabkins solution in an appropriate tube, with direct reading on the portable hemoglobinometer Agabe^®^ (validated by Exa-M Instrumentação Biomédica LTDA., Mogi das Cruzes, SP, Brazil) by the cyanomethaemoglobin method. Before blood collection, parents/guardians were asked about the presence of genetic hemoglobinopathies in their children, with no notifications regarding this item. Children who showed infection or inflammation did not participate in the research while they were ill. Anemia was defined by the concentration of Hb <11.0 g/dL for children under five years of age.^1^

Through a questionnaire, demographic and socioeconomic information were obtained: sex, age, child’s weight at birth, maternal schooling and occupation, family income, participation in social programs, inclusion of the family in the FHS and use of iron supplementation offered by the National Program of Iron Supplementation.

Statistical analyzes were performed using the Statistical Package for the Social Sciences (SPSS) software, version 20.0 for Windows (IBM, New York, United States). The Kolmogorov-Smirnov test was used to verify the normality of continuous variables. Initially, the high and low vulnerability regions were characterized using the χ^2^ test and the medians for Hb concentration were calculated using the Mann-Whitney test, as well as contingency tables for anemia prevalence.

Next, multiple regression analysis was carried out. The concentration of Hb was identified as a dependent variable, and the independent variable was the region of the day care center being defined by the child’s vulnerability and demographic conditions, and by the family’s socioeconomic conditions. The variables that presented p<0.20 in the univariate analysis were included in the multiple modeling process, considering associations with p<0.05 as significant in the regression model.

The research was approved by the Research Ethics Committee of the School of Public Health of Universidade de São Paulo (CEP/FSP - USP), no. 773.287, according to ethical standards. The guardians of the children authorized data collection by signing the informed consent. Children diagnosed with anemia were referred to the Pediatric Clinic of the University Hospital of Taubaté for proper detailed investigation of the etiology and appropriate treatment of anemia.

## RESULTS

A total of 363 children aged between 24 and 48 months participated in this study, all of whom attended public day care centers in the part-time (morning/afternoon period) or full-time programs. There were no differences between children in the investigated regions in terms of sex or age, however, there was a polarization of adverse socioeconomic conditions for vulnerable regions (Table 1).

**Table 1:** Characterization and association of socioeconomic regions of day care centers with demographic and socioeconomic variables, Taubaté, 2014*.

Variables	Total		Region of day care center		p-value**
	n	%	Vulnerable % (n)	Non-vulnerable % (n)	
Sex					
Male	169	46.6	62.7 (106)	37.3 (63)	0.638
Female	194	53.4	60.3 (117)	39.7 (77)	
Age					
24-35 months	141	38.8	60.3 (85)	39.7 (56)	0.720
36-48 months	222	61.2	62.2 (138)	37.8 (84)	
Weight at birth					
<2,500 g	39	11.3	56.4 (22)	43.6 (17)	0.528
≥2,500 g	305	88.7	61.6 (88)	38.4 (117)	
Maternal schooling					
≤5 years of schooling	46	13.0	71.7 (33)	28.3 (13)	<0.001
6-9 years of schooling	89	25.1	73.0 (65)	27.0 (24)	
10-12 years of schooling	144	40.7	61.1 (88)	38.9 (56)	
≥13 years of schooling	75	21.2	38.7 (29)	61.3 (46)	
Maternal work					
Yes	268	74.4	57.1 (153)	42.9 (115)	0.004
No	92	25.6	73.9 (68)	26.1 (24)	
Family income					
<1 MW***	43	12.1	76.9 (33)	23.3 (10)	0.003
1 to 2 MW	139	39.2	67.6 (94)	32.4 (45)	
2 to 3 MW	104	29.3	56.7 (59)	43.3 (45)	
≥3 MW	69	19.4	46.4 (32)	53.6 (37)	
Governmental Aid					
Yes	103	28.4	72.8 (75)	27.2 (28)	0.005
No	260	71.6	56.9 (148)	43.1(112)	
Family Health Strategy					
Yes	129	37.0	82.2 (106)	17.8 (23)	<0.001
No	220	63.0	46.8 (103)	53.2 (117)	
Access to iron supplement					
FHS/SUS	55	53.4	74.5 (41)	25.5 (14)	0.001
Bought at drugstores	48	46.6	43.8 (21)	56.3 (27)	
Day care program					
Part-time	184	50.7	65.2 (120)	34.8 (64)	0.133
Full-time	179	49.3	57.5 (103)	42.5 (76)	

*Totals differ due to losses in variables; **χ^2^ test; ***BRL 723 or USD 306, in 2014; MW: minimum wage; FHS: Family Health Strategy; SUS: Unified Health System.

The vulnerable population was associated with low maternal schooling, low family income, maternal work outside the household, beneficiaries of social programs, greater number of families with access to the FHS and the National Iron Supplementation Program (p<0.05).

Anemia was present in 19.3% of the children studied. Regarding the location of the day care centers they attended, there was a statistical difference in the medians of Hb concentration: day care centers with high socioeconomic vulnerability had more anemic children than those in non-vulnerable regions. An even lower concentration of Hb was observed in boys and children of mothers with less schooling (p<0.05), as shown in Table 2.

**Table 2:** Association of hemoglobin concentration (Hb) and prevalence of anemia with demographic and socioeconomic variables, Taubaté, 2014*.

Variables	n	[Hb] g/dL** Median	Prevalence of anemia^†^	
			%	n
Sex				
Male	169	12.9	21.9	37
Female	194	13.4	17.0	33
		p=0.031		p=0.239
Age				
24-35 months	141	13.1	22.7	32
36-48 months	222	13.0	17.1	38
		p=0.775		p=0.189
Weight at birth				
<2,500 g	39	13.6	10.3	4
≥2,500 g	305	13.0	20.3	62
		p=0.346		p=0.133
Maternal schooling				
≤9 years of schooling	135	12.7	21.2	35
>9 years of schooling	219	13.2	17.5	31
		p=0.028		p=0.387
Maternal work				
Yes	268	13.0	20.5	55
No	92	13.6	15.2	14
		p=0.147		p=0.265
Family income				
≤2 MW^‡^	182	13.0	21.4	39
>2 MW	173	13.1	16.8	29
		p=0.648		p=0.264
Governmental Aid				
Yes	103	13.4	17.5	18
No	260	13.0	20.0	52
		p=0.242		p=0.583
Region of day care center				
Vulnerable	223	12.9	22.0	49
Non-vulnerable	140	13.3	15.0	21
		p=0.028		p=0.101
Day care program				
Part-time	184	50.7	22.8	42
Full-time	179	49.3	15.6	28
		p=0.270		p=0.283
Total	363	13.1	19.3	70

*Totals differ due to losses in variables; **Mann-Whitney test; ^†^χ^2^ test; ^‡^BRL 1,446, or USD 612, in 2014; MW: minimum wage.

In the multiple regression model, it was shown that only the day care center region variable remained in the model, since high socioeconomic vulnerability has greater explanatory power for iron deficiency anemia in this population than the other independent variables. Thus, attending a day care center in a socially and economically vulnerable region was the variable in which preschoolers had the lowest Hb concentrations (Table 3).

**Table 3:** Multivariate regression model among the determinants of hemoglobin concentration in preschoolers, Taubaté, 2014.

Variable	Nonstandardized coefficients		Standardized coefficients	95%CI		p-value
	B	Standard model	Beta*	Lower limit	Upper limit	
Constant	10.883	0.588		9.727	12.040	0.000
Day care in vulnerable region**	0.576	0.223	0.138	0.137	1.015	0.010
Maternal work	0.517	0.251	0.110	0.024	1.009	0.050
Male sex	0.383	0.216	0.093	-0.042	0.807	0.077

*Multivariate regression, model adjusted for maternal education; **p<0.05.

## DISCUSSION

The prevalence of anemia found in the city of Taubaté is classified as a mild to moderate public health problem,^1^ possibly reflecting access to health and education for the municipality’s population. In this way, this sensitive group responds better to the good conditions of basic public services that serve almost the entire population. This condition is justified by the high access of children to day care centers, regardless of the family’s socioeconomic region, by the greater number of years of maternal schooling and by the fact that low-income groups are the biggest beneficiaries of income transfer and basic health care programs, as characterized by the population.

Analyzes of socioeconomic vulnerability on the prevalence of anemia in preschoolers are current and relevant, since the factors that influence the prevalence of anemia are not only related to income, but rather to the complex set of variables that act in isolation or in clusters on the development of this condition, which makes it necessary to use other indicators in the investigation of vulnerable population groups.^10^ These indicators, in a simplified way, have already been used in some studies,^12^ ^,^ ^13^ as the unfavorable conditions in the economic and social levels of the family are part of the distal and proximal determinants of childhood anemia.^14^

The prevalence of anemia found according to age groups corroborate those observed for Brazil, with 21.7% for children aged 24 to 35 months and 19.6% for those aged 36 to 59 months.^6^ This is explained by the inverse relationship between age and the prevalence of anemia: children between the ages of two and four present a different situation regarding the risk of iron deficiency when compared to infants, since they are no longer exposed to the great demands of iron resulting from the high speed of growth seen especially in the first two years of life. In addition, the dietary intake of children over two years of age is no longer based on breast milk, being similar to adult eating habits in composition and consistency, which reflects a consumption of greater amounts of source foods, as well as foods fortified with iron, which translates into the decrease in prevalence found in studies that analyzed the presence of anemia in childhood.^6^ ^,^ ^15^

When comparing the percentage of childhood anemia in Brazil, there is a different situation from other developing countries, such as Vietnam, Benin and Mali, with prevalence rates of 45.1, 82 and 83%, respectively.^16^ ^,^ ^17^ The same occurs in different regions of Brazil: cities with different HDIs exhibit high prevalence of nutritional deficiencies.^18^ This inconsistency in global and regional situations can be justified by the “pan-social” character of iron deficiency that affects rich and poor countries,^1^ or families from different income strata within the same country or region.^14^

In accordance with our findings, in the state of Pernambuco, Northeastern Brazil, a decrease in the prevalence of anemia was reported for children older than 24 months of age within a decade, with the economic index being the only one that remained associated with anemia.^12^

In this sense, despite these considerations, it is noteworthy that the level of Hb concentration is significantly lower in children attending day care centers in regions of high socioeconomic vulnerability. This is possibly due to the numerous and complex coexistence factors in the etiology of anemia. Even though Brazil has improved the situation of malnutrition, basic sanitation and child morbidity,^19^ differences still persist, conditioned by the high inequality in the distribution of income in the strata of society.

In the city of Taubaté, although the Gini coefficient, used to measure inequality in income distribution, is better than that of the national context,^20^ socioeconomic inequality is present in the assessed population. In this perspective, Barata et al.^21^ consider that, through the analysis of family income and education, it is possible to make a direct association with the health status of individuals, in addition to allowing the definition of a framework of social vulnerability.

Thus, the division of day care centers according to their location in regions distributed by socioeconomic vulnerability, as a synthesis variable, was explanatory for the different concentrations of Hb. This result is in line with the literature, given that populations most susceptible to anemia live on the periphery of urban centers, suffer from the lack of job opportunities, low wages and poor housing, education and health conditions.^14^ This finding, however, was not described by Bueno et al.^22^ when they assessed the prevalence of anemia among children in public day care centers in the various administrative divisions of the city of São Paulo, possibly because it is a population with homogeneous socioeconomic characteristics.

Consequently, research aimed at families that suffer socioeconomic inequality is important, as children in situations of social inequity have higher prevalence of anemia because they do not alleviate poor child health conditions among upper and middle social classes, as is the case in population-based studies that mitigate the prevalence rates of nutritional deficiencies of vulnerable populations with those of economically non-vulnerable populations.^18^

The disproportionality of populations of low socioeconomic status who suffer more from iron deficiency, especially in developing countries, points to flaws in strategies to control anemia worldwide.^23^ Watchful of this, the World Health Organization (WHO) recommended more specific measures, with a view to putting pressure on developing countries in the search for alternatives to control anemia,^24^ thus promoting a great debate on the development of new strategies against nutritional deficiencies globally.

In Brazil, even with the implementation of a strategy to control iron deficiency, considered the most effective - fortification of wheat and corn flours -, the prevalence of anemia remains especially high among the population with lower socioeconomic status, possibly due to problems in public policies and the absence of actions to control this nutritional deficiency in the more than 10 years following the implementation of this strategy. The unmet expectation in the control of iron deficiency may be a consequence of low consumption and low bioavailability of the iron used in fortified products^25^ or even failures in the coverage and adherence of iron supplementation in health units and the absence of adequate guidance services.^26^

Thus, taking into account the latest WHO guidance, which indicates the use of multiple micronutrients in powder form for homemade fortification as an alternative,^24^ a new intervention was formulated by the Brazilian government in partnership with national and international institutions, named NutriSUS, which aims to improve iron status and reduce anemia in children aged between 6 and 23 months.^27^ Even knowing the effectiveness of this interventionist measure,^28^ the real effectiveness has not yet been evaluated, since the implementation process is recent. However it can be said in a simplistic way that this is another superficial action to solve the issue of low-income families, victims of childhood anemia, as interventions that do not deal with the proximal determinants of anemia are aimed at the same problems faced by existing interventionist strategies. After all, it is known that the main measure that definitively minimizes the risk of anemia is the diet with the best iron potential.

As was found in the results of this study, since the service is similar in all preschool units in the city of Taubaté, regardless of the location of the day care center, it can be assumed that the difference in family feeding practices is the main determinant of iron deficiency, since children are given the same school lunch. This point brings to the discussion a fundamental aspect hitherto presumed, diet as a determinant of health.

In this context, a more in-depth analysis on the topic involves the human right to adequate food, which protects both the health and the dignity of a population. According to General Comment No. 12 of the Committee on Economic, Social and Cultural Rights, the human right to adequate and healthy food for everyone and at all times is not restricted to the minimum number of calories required per day, but integrates a set of rights related to social, economic, cultural, climatic, ecological adequacy, among others.^29^

Brazil has made progress in this regard by creating the National System of Food and Nutritional Security to monitor and evaluate the nutritional situation of the population and act jointly in the formulation and implementation of policies and actions to fight hunger.^30^ However, it is known that many structural challenges permeate the guarantee of the right to food and health, the main promoters of justice and social equity. In addition, one cannot ignore the fact that the consequences of anemia for the country affect not only children, but the entire population. Its high indirect cost in reducing the work capacity due to shortages in the employment of men and women,^2^ maternal mortality, prematurity, low weight of children at birth and, above all, the possible consequences on cognitive development that occur in childhood and translate into failure and dropping out of school over the course of life, imposing, in the future, a substantial burden on the country’s economy.^3^

In another scenario of discussion, when the prevalence of anemia related to the birth weight of these children is observed, it is clear that those with low weight at birth had a lower risk than those with higher weight at birth (>2500 g). Even if not statistically significant, it may indicate that the care provided to children with low weight at birth may have protected them from childhood anemia. It is known that such children should receive differentiated assistance in health services, as well as by family members, involving the monitoring of biological, social and affective aspects.

Anemia, being a pathology that does not indicate specific signs or symptoms, is not valued by the population or even by the health teams that monitor apparently healthy children. Therefore, it is recommended that health services work through food and nutrition education to raise the population’s awareness of the risks arising from iron deficiency and the deleterious consequences that lead to quality of life.

Although the cross-sectional study model may have limitations as it does not achieve a definite cause-and-effect relationship like longitudinal research, it allows reflection on the situational event and the status quo, being an adequate instrument for formulating hypotheses that can be investigated with new studies.

Thus, it was observed that a lower concentration of Hb in preschoolers is associated with the location of day care centers in a region of socioeconomic vulnerability, since children who attend such day care centers face adverse family conditions such as low income, working mothers with low schooling, although they receive social benefits and health monitoring by the FHS and the National Iron Supplementation Program.

The high HDI of the studied municipality does not seem to have the expected effectiveness in the fight against anemia, even with favorable life indicators, since the minimally unfavorable socioeconomic conditions of the family are still responsible for lower Hb concentrations in children. In view of the analysis of the most proximal factor of nutritional deficiency, which is diet, it can be assumed that when equal conditions are offered in the provision of school lunches in day care centers, the most appropriate public intervention to minimize the risk of childhood anemia in the population is the definitive improvement of family eating conditions, with increased income and social benefits, expansion of health strategies and services, as well as maternal work and schooling conditions.
